# Pyroptosis in inflammatory diseases and cancer

**DOI:** 10.7150/thno.71086

**Published:** 2022-05-16

**Authors:** Zhiping Rao, Yutong Zhu, Peng Yang, Zhuang Chen, Yuqiong Xia, Chaoqiang Qiao, Weijing Liu, Hongzhang Deng, Jianxiong Li, Pengbo Ning, Zhongliang Wang

**Affiliations:** 1 Engineering Research Center of Molecular & Neuroimaging, Ministry of Education, School of Life Science and Technology, Xidian University, Xi'an, Shaanxi, 710126, P. R. China; 2 Academy of Advanced Interdisciplinary Research, Xidian University, Xi'an, Shaanxi, 710071, P. R. China; 3 Department of Radiotherapy, Chinese PLA General Hospital, Beijing, 100071, P. R. China.

**Keywords:** Pyroptosis, signaling pathway, gasdermin, inflammatory diseases, cancer

## Abstract

Pyroptosis is a lytic and inflammatory type of programmed cell death that is usually triggered by inflammasomes and executed by gasdermin proteins. The main characteristics of pyroptosis are cell swelling, membrane perforation, and the release of cell contents. In normal physiology, pyroptosis plays a critical role in host defense against pathogen infection. However, excessive pyroptosis may cause immoderate and continuous inflammatory responses that involves in the occurrence of inflammatory diseases. Attractively, as immunogenic cell death, pyroptosis can serve as a new strategy for cancer elimination by inducing pyroptotic cell death and activating intensely antitumor immunity. To make good use of this double-edged sword, the molecular mechanisms, and therapeutic implications of pyroptosis in related diseases need to be fully elucidated. In this review, we first systematically summarize the signaling pathways of pyroptosis and then present the available evidences indicating the role of pyroptosis in inflammatory diseases and cancer. Based on this, we focus on the recent progress in strategies that inhibit pyroptosis for treatment of inflammatory diseases, and those that induce pyroptosis for cancer therapy. Overall, this should shed light on future directions and provide novel ideas for using pyroptosis as a powerful tool to fight inflammatory diseases and cancer.

## Introduction

The body maintains a dynamic balance between cell proliferation and cell death, which plays a significant role in the physiopathological processes of multicellular organisms. Cell death is usually categorized as non-programmed cell death and programmed cell death (PCD). Pyroptosis is a type of inflammatory PCD. In 1992, researchers discovered that mouse macrophages infected with *Shigella flexneri* eventually underwent cell death 1. Later, researchers revealed that inflammatory caspase-1 was activated during *Shigella flexneri*- or *Salmonella*-induced cell death 2, 3. So, this type of cell death was originally considered as caspase-dependent apoptosis. However, in 2001, Cookson et al. found that *Salmonella*-induced cell death displayed completely different characteristics from those of apoptosis. Apoptotic cells have intact membranes accompanied by cell shrinkage, while the membrane integrity of *Salmonella*-infected macrophages is destroyed by cell swelling 4, 5. Hence, a new term, pyroptosis, was proposed to describe this type of cell death 5, which is characterized by cell membrane pore formation, membrane rupture, cell swelling, and release of cell contents. The factors released during cell death, such as interleukin-1β (IL-1β) and interleukin-18 (IL-18), amplify the inflammatory effects and activate immune responses 6, 7.

Although pyroptosis has been proposed for a long time, the underlying mechanism was only uncovered in 2015 upon the discovery and identification of gasdermin D (GSDMD) protein. It was found that cleavage of GSDMD by caspase-1 results in the release of its N-terminal domain (GSDMD-NT), which then forms pores in the cell membrane, thus demonstrating that GSDMD is the central executor of pyroptosis 8. In addition to GSDMD, gasdermin family includes five other members. The human gasdermin family comprises of GSDMA, GSDMB, GSDMC, GSDMD, GSDME/DFNA5, and PVJK/DFNB59. In mice, there are five gasdermin members, including GSDMA, GSDMC, GSDMD, GSDME, and PJVK/DFNB59, but no GSDMB 9, 10. All gasdermins except DFNB59 have two conserved domains, an N-terminal effector domain and a C-terminal inhibitory domain 11. In general, binding of the C-terminal inhibits the pore-forming activity of the N-terminal. In the presence of numerous microbes or other stimulations, gasdermin is cleaved by active caspases or granzymes to liberate the N-terminal domain, which forms large pores in the membrane to release cell contents and execute pyroptosis 12. The Ragulator-Rag-mTORC1 pathway is required for GSDMD oligomerization and pore formation in macrophages 13. Cell-surface protein NINJ1 has an essential role in the induction of plasma membrane rupture, which is responsible for releasing intracellular molecules that propagate the inflammatory response 14. However, the membrane pore can be repaired by endosomal sorting complex required for transport machinery, which initiates by calcium influx through GSDMD pores 15. The membrane repair can allow cells to restrict pyroptosis and provide insight into cellular survival mechanisms during pyroptosis.

The occurrence of pyroptosis often crosstalk with a variety of cell death such as apoptosis and necroptosis. Although these different types of cell death induced by distinct mechanisms, they share some similarities and could be activated alone or simultaneously under different conditions. During apoptosis, cleavage of GSDME by caspase-3 mediates progression to pyroptotic cell death 16. Apoptotic caspase-8, generally correlated to apoptosis, was shown to cleave GSDMD and induce pyroptosis 17. In turn, the inflammatory caspase-1 could activate apoptosis in the absence of GSDMD. This caspase-1-induced apoptosis depends on caspase-3 and involves caspase-9 18. Crosstalk between necroptosis and pyroptosis was also discovered recently. Mixed-lineage kinase domain-like protein, the executioner of necroptosis, can also activate NLRP3 inflammasome to promote the maturation of IL-18 and IL-1β 19. However, the maturation and release of cytokines are independent of GSDMD from necroptotic cells 20.

**Figure 1:**
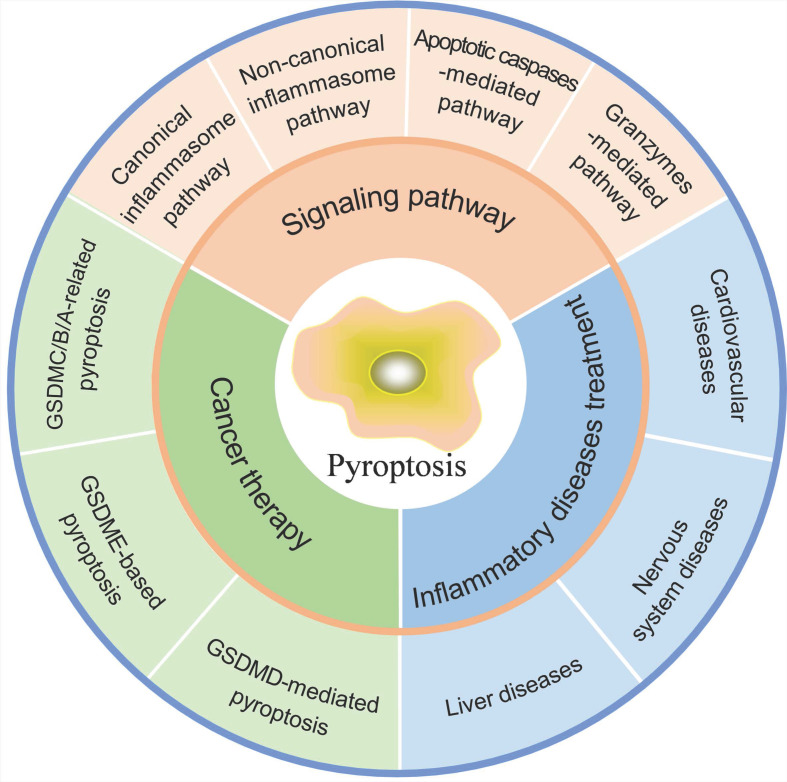
Pyroptosis in inflammatory diseases and cancer.

Normally, moderate pyroptosis contributes to host defense against pathogen infection, but excessive pyroptosis leads to intemperate inflammatory responses, massive cell death, and serious tissue damage, causing inflammatory or autoimmune diseases. Meanwhile, as a pro-inflammatory type of cell death, pyroptosis paves a new way for cancer elimination by activating antitumor immune response. Here, we first demonstrate different signaling pathways of pyroptosis to gain deep insight into molecular mechanisms. Next, the functions and therapeutic applications of pyroptosis in inflammatory diseases are discussed. Finally, we summarize the roles of pyroptosis in cancer and recent progress in strategies that induce pyroptosis for cancer therapy (Figure 1), which will point out the direction for future research.

## Signaling pathways of pyroptosis

**Figure 2:**
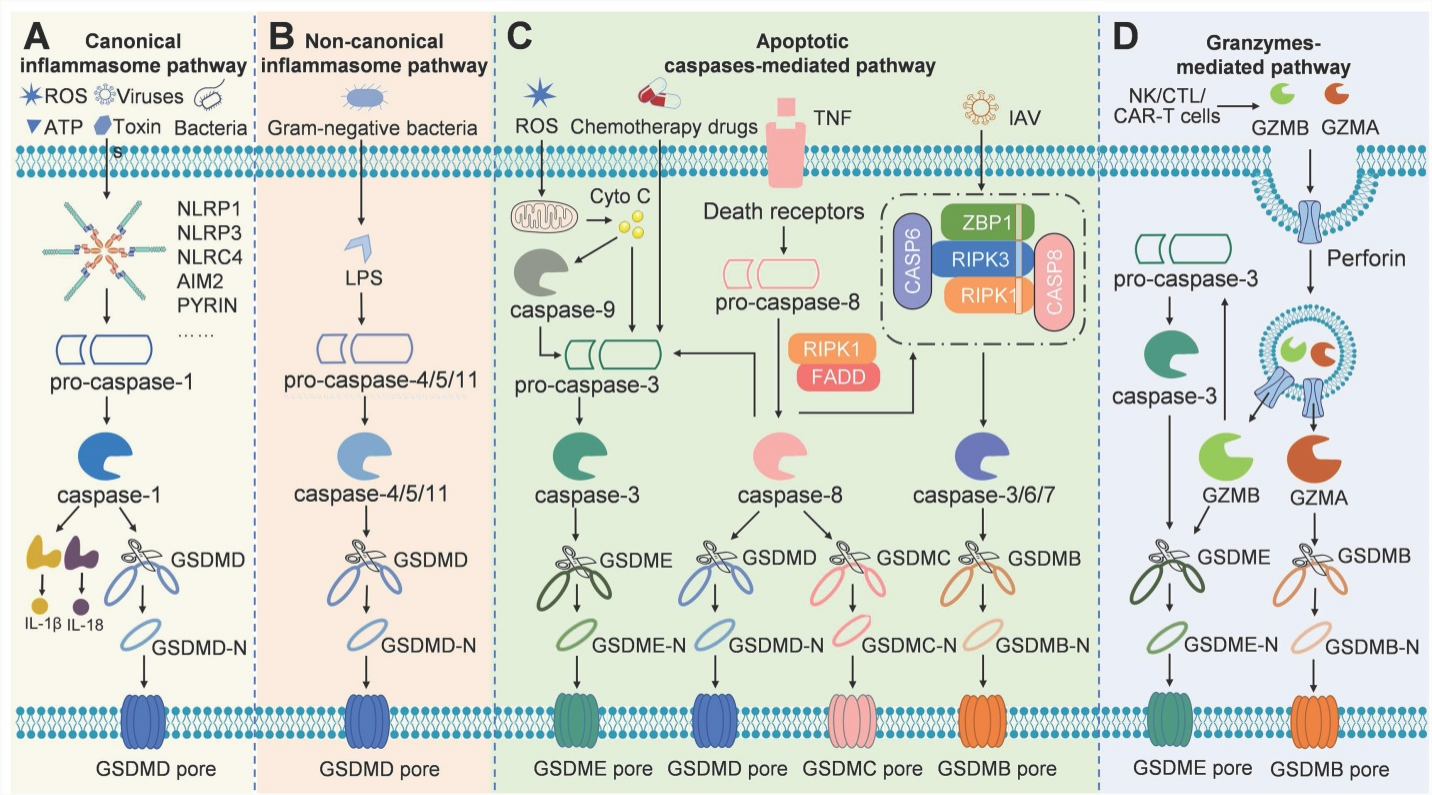
Schematic illustration of the different pyroptosis pathways. (A) In the canonical inflammasome pathway, pathogen-associated molecular patterns or damage-associated molecular patterns like viruses, bacteria, toxins, ATP, or ROS stimulates inflammasome, which then activates caspase-1 to cleave GSDMD for pore formation. (B) LPS from Gram-negative bacteria activates caspase-4/5/11 directly, followed by GSDMD cleavage to execute pyroptosis in the non-canonical inflammasome pathway. (C) Apoptotic caspases-mediated pyroptosis pathway can be engaged through mechanisms such as caspase-3/GSDME, caspase-8/GSDMC, caspase-6/GSDMB, and so on. (D) In the granzymes-mediated pathway, GZMA or GZMB derived from cytotoxic lymphocytes can cleave GSDMB or GSDME respectively for pore formation and pyroptosis.

At present, there are mainly four distinct signaling pathways that have been identified to induce pyroptosis, including canonical and non-canonical inflammasome pathways, apoptotic caspases-mediated pathway, and granzymes-based pathway (Figure 2). In these signaling pathways, gasdermin proteins are the final executioners, which need to be cleaved by upstream caspases or granzymes. Caspases can be categorized into inflammatory and apoptotic caspases based on function 21. Commonly, caspases-1/4/5/11 belong to inflammatory caspases, which play key roles in the innate immune response by inducing pyroptosis to interrupt replication of invading pathogens, and by processing pro-inflammatory cytokines to maturation and release 22. Activation of inflammatory caspase provides the first line of defense against infectious pathogens. Caspase-1 is activated in a multiprotein complex called the inflammasome in the canonical pyroptosis pathway. The inflammatory caspase-4/5/11 do not need such molecular complex for their activation, which were shown to bind lipopolysaccharide (LPS) directly. Apoptotic caspases function predominantly to initiate and execute apoptosis. Recent studies have shown that they can serve as the proteases to cleave gasdermins for pyroptosis induction 16. The details of each signaling pathway of pyroptosis are discussed below.

### Canonical inflammasome pathway

The canonical inflammasome pathway was the first to be discovered. Inflammasomes are multi-protein complexes assembled in response to pathogen-associated molecular patterns or non-pathogen-related damage-associated molecular patterns. Generally, inflammasomes are comprised of intracellular pattern recognition receptors (PRRs), apoptosis-associated speck-like protein containing a caspase-recruitment domain (ASC), and inflammatory caspases 23. The most common PRRs include nucleotide-binding oligomerization domain-like receptors (NLRs, including NLRP1, NLRP3, and NLRC4), absent in melanoma 2 (AIM2), and pyrin 24, 25 (Figure 2A). NLRP1 is composed of an N-terminal pyrin domain (PYD), a nucleotide-binding oligomerization domain (NOD), a leucine-rich repeats (LRR), and a C-terminal caspase recruitment domain (CARD) 26. The PYD is required for combining with ASC. NOD involves in adenosine triphosphate (ATP)-dependent activation of the signal. LRR is responsible for ligand recognition and auto-inhibition. CARD takes part in pro-caspase-1 recruitment. Anthrax lethal toxin, muramyl dipeptide, and components of *Toxoplasma gondii* can activate NLRP1 27. NLRP3 consists of an N-terminal PYD, a NOD, and an LRR, without C-terminal CRAD. The NLRP3 is activated by various factors, including bacteria, viruses, fungi, uric acid, reactive oxygen species (ROS), adenosine triphosphoric (ATP), and endogenous damage signals 28. Extracellular ATP induces IL-1β secretion and caspase-1 activation by activating the P2X purinoreceptor 7 (P2X7) and inducing K^+^ efflux 29. NLRC4 has an N-terminal CARD domain, a central NBD domain, and a C-terminal LRR domain. NLRC4 responds to type III secretory system proteins and flagellin 30. AIM2 holds a PYD domain and a DNA-binding HIN-200 domain that can sense bacteria- or viruses-derived double-stranded DNA 31. Pyrin has a PYD domain, two B-boxes, and a C-terminal SPRY/PRY domain. Pyrin mainly recognizes the inactivating modifications of host Rho guanosine triphosphatases mediated by various bacterial toxins or effectors 25. Upon stimulation of PRRs, pro-caspase-1 is recruited directly by CARD-carrying PRRs or indirectly *via* ASC to assemble caspase-1-dependent inflammasomes, which is followed by caspase-1 activation through self-cleavage. Active caspase-1 not only cleaves inactive IL-1β and IL-18 precursors, but also cleaves GSDMD to release GSDMD-NT for pore-formation, eventually leading to inflammatory responses and pyroptosis 32. The canonical inflammasome pathway-mediated pyroptosis mainly occurs in immune cells and serves as a host defense mechanism against pathogen infection.

### Non-canonical inflammasome pathway

The non-canonical inflammasome pathway is independent of the classical inflammasome complex. Most Gram-negative bacteria activate the non-canonical inflammasome pathway. Extracellular LPS can induce the expression of type I interferon, which then forms a feedback loop and activates type I interferon receptor to induce caspase-11 expression 33, 34. Vacuolar Gram-negative bacteria release their LPS into the cytosol through vacuolar rupture triggered by interferon-inducible guanylate-binding proteins. The released LPS can directly bind to and activate caspase-11, which then cleaves GSDMD to promote pyroptosis 35, 36 (Figure 2B). In human, caspase-4/5 can be activated by intracellular LPS. Caspase-4/5/11 cannot cleave pro-IL-18 and pro-IL-1β directly, but K^+^ efflux caused by GSDMD-NT pores can activate NLRP3 and caspase-1, eventually leading to maturation and release of IL-18 and IL-1β 37. In addition, Yang et al. demonstrated that cleavage of the pannexin-1 channel and ATP release occur in a caspase-11-dependent manner upon LPS stimulation, which then activate ATP-gated ion channel P2X7, ultimately resulting in K^+^ efflux and subsequent NLRP3/caspase-1 activation in bone marrow-derived macrophages 38. Therefore, the activation of NLRP3 inflammasome induced by the active caspase-11 is required for IL-1β processing in the non-canonical inflammasome pathway.

### Apoptotic caspases-mediated pathway

In addition to inflammatory caspase-1/4/5/11, some apoptotic caspases can also trigger pyroptosis (Figure 2C). Chemotherapy drugs can induce caspase-3-mediated apoptosis, if the target cells express GSDME, the activated caspase-3 can cleave GSDME to induce pyroptosis, which switches the mode of cell death. Wang et al. found that cisplatin and other conventional chemotherapy drugs can induce pyroptosis through caspase-3-mediated cleavage of GSDME 39. Another apoptotic caspase that can trigger pyroptosis is caspase-8, which can induce the cleavage of GSDMD to elicit pyroptosis during Yersinia infection 17, 40. When transforming growth factor-β-activated kinase 1 is inhibited by the Yersinia effector YopJ, lysosome Rag-Ragulator served as a platform for activating a Fas-associated death domain/receptor-interacting serine-threonine protein kinase 1/caspase-8 complex to trigger pyroptosis 41. Besides, caspase-8 can also cleave GSDMC, liberating the N-terminus of GSDMC to form pores in the cancer cell membrane 42. In addition, Chao et al. showed that apoptosis-related caspase-3/6/7 cleaves GSDMB, thus removing the C-terminal repressor domain, to cause the release of the N-terminal effector domain, which perforates the cell membrane and ultimately evokes cell pyroptosis 43.

### Granzymes-mediated pathway

Recently, studies have shown that natural killer cells, cytotoxic T lymphocytes, or chimeric antigen receptor T cells derived granzymes, which are delivered by perforin into target cells, can cleave specific gasdermin family members to induce cancer cell pyroptosis (Figure 2D). Granzyme A (GZMA) is the most abundant serine protease of the granzyme family, which has traditionally been recognized as a mediator of cell death. However, there are many reports have shown that GZMA fails to kill target cells in vitro unless very high concentrations are used 44-46. Accumulating evidence now suggests the role of GZMA in modulating inflammation, such as inducing the maturation and release of pro-inflammatory cytokines 47-49. Pyroptosis, one type of cell death that is accompanied by pro-inflammatory cytokines release, may be associated with GZMA. Recently, Zhou et al found that GZMA derived from cytotoxic T lymphocytes cleaves GSDMB to form pores in the membrane, resulting in pyroptosis of GSDMB-expressing cancer cells 50. So, whether the GZMA can kill cancer cells through pyroptosis also depends on the expression of GSDMB, which do not express in some human tissue and is absent in mouse. Natural killer cell-derived granzyme B (GZMB) can directly cleave GSDME at the same site that is cleaved by caspase-3, leading to the release of the effector N-terminal, which perforates the cell membrane 51. GZMB induces GSDME-dependent pyroptosis in tumor targets both directly by cleaving GSDME and indirectly by activating caspase-3. The direct cleavage of GSDME by GZMB provides a simple mechanism and pathway for triggering inflammatory death. Caspase-resistant cancer cells should be susceptible to this direct pathway, provided that the cancer cells express GSDME. Granzymes-mediated cancer cell pyroptosis may amplify the inflammatory response in the tumor microenvironment (TME), thereby recruiting more immune cells for antitumor immunity.

## Inhibiting pyroptosis to treat inflammatory diseases

In normal physiology, moderate pyroptosis plays an important role in the host defense against pathogenic microorganisms 52, 53. However, dysregulated inflammatory response and cell death caused by overactivated pyroptosis may be involved in the pathological progression of many diseases 54, 55, especially inflammatory diseases. Herein, we mainly discuss the role and therapeutic potential of pyroptosis in inflammatory diseases, like cardiovascular diseases, liver diseases, and nervous system diseases.

### Cardiovascular diseases

Cardiovascular diseases are the primary cause of patient suffering and high mortality worldwide. Recently, many studies have shown that pyroptosis is closely related to the occurrence and development of cardiovascular diseases, such as atherosclerosis, ischemia-reperfusion injury (IRI), and myocardial infarction (MI).

The pathogenesis of atherosclerosis involves smooth muscle cell proliferation and migration, endothelial cell dysfunction, pro-inflammatory cytokine secretion, and cell death 56. Previous studies have illustrated that pyroptosis in macrophages, endothelial cells, and smooth muscle cells are related to the progression of atherosclerosis 57. Duewell et al. showed that cholesterol crystals can activate caspase-1 through the NLRP3 inflammasome, which cleaves pro-IL-18 and pro-IL-1β to produce their mature forms, resulting in inflammation and atherosclerosis formation 58.

IRI involves different types of cell death, among which pyroptosis is one of the commonly observed cell death modes. Lou et al. illustrated that microRNA (miR)-424 is markedly upregulated in IRI conditions, which reduces the expression of cysteine-rich secretory protein LCCL domain-containing 2 and results in the upregulation of caspase-1, IL-18, and IL-1β in cardiac pyroptosis under IRI 59.

**Figure 3:**
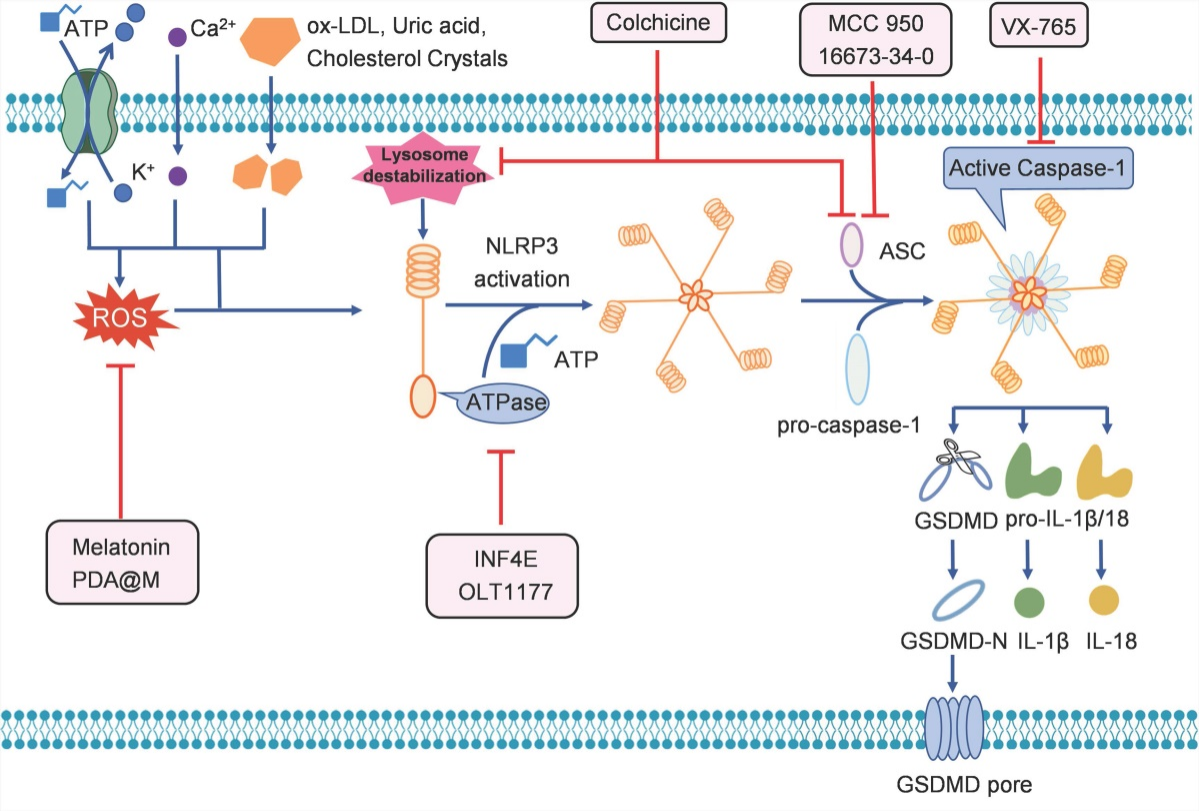
Potential strategies targeting pyroptosis for the treatment of cardiovascular diseases.

Since pyroptosis is involved in the occurrence and progression of cardiovascular diseases, many strategies have been developed to target pyroptosis for the treatment of these diseases (Table 1, Figure 3). There are numerous inhibitors of NLRP3 inflammasome, such as INF4E and OLT1177, when given in mouse model of IRI, the inhibitors significantly reduce infarct size by inhibiting the ATPase activity of NLRP3 60, 61. The small molecule 16673-34-0 prevents NLRP3 oligomerization in cardiomyocytes and limits myocardial injury after myocardial ischemia-reperfusion in the mouse model 62. MCC950 is shown to inhibit NLRP3-induced ASC oligomerization, by which reducing infarct size, improving cardiac remodeling, and preventing left ventricular dysfunction in a pig model of MI 63. Colchicine acts upstream of NLRP3 to block the opening of P2X7 channel and interfere with ASC polymerization 64, 65. Treatment with colchicine successfully attenuates NLRP3 inflammasome activity, improves cardiac function, and prolongs survival after MI 66.

Besides the inhibitors that directly affect NLRP3, there are agents that can suppress the activity of NLRP3 indirectly. Zhang et al. showed that the anti-inflammatory agent melatonin can prevent endothelial cell pyroptosis by regulating the signaling pathway of maternally expressed gene 3/miR-223/NLRP3 in atherosclerosis 67. Wang et al. showed that melatonin reduced cigarette smoke extract-induced pyroptosis by inhibiting the ROS/NLRP3 axis in atherosclerosis 70. Liraglutide alleviates NLRP3 inflammasome-mediated pyroptosis in H9c2 cells, by regulating the sirtuin 1 (SIRT1)/NAPDH oxidase 4/ROS pathway 71. Wei et al. reported that a polydopamine-based biomimetic nanoplatform (PDA@M) can inhibit pyroptosis to protect the myocardium against IRI. PDA@M consists of a polydopamine core and a macrophage membrane shell, to achieve site-specific antioxidative efficacy 68. The results demonstrated that PDA@M targets the infarcted myocardium to suppress the NLRP3/caspase-1 pathway, thus exerting antioxidative and antipyroptosis functions, suggesting that it may serve as a potential therapeutic agent for IRI.

Caspase-1 inhibitors can also inhibit pyroptosis, and thus, serve to be useful in the treatment of cardiovascular diseases. For example, the caspase-1 inhibitor VX-765 has been shown to produce a sustained reduction in myocardial infarct size and facilitate preservation of ventricular function in a pre-clinical model of IRI treated with a P2Y_12_ receptor antagonist 69. Moreover, VX-765 was able to reduce myocardial infarction in a model of IRI, demonstrating that caspase-1 inhibition is an effective method for treating pyroptosis-triggered cardiovascular diseases 72.

### Liver diseases

Liver diseases are serious problems that endanger human health worldwide. Recently, studies have demonstrated that pyroptosis is responsible for the progression of liver diseases. When the intestinal flora is out of balance, the gut microflora can enter the liver through the intestine-liver axis, which then triggers pyroptosis in liver cells 73.

Non-alcoholic fatty liver disease (NAFLD) has become a serious health problem owing to its high incidence and high risk of cirrhosis. The roles of cell necrosis and apoptosis in NAFLD have been emphasized, but it has only recently been recognized that pyroptosis may also play an important role in this condition. NAFLD is further categorized into non-alcoholic fatty liver (NAFL) and non-alcoholic steatohepatitis (NASH). NAFL is characterized by the accumulation of triglycerides in hepatocytes, while NASH involves massive cell damage, inflammatory cell infiltration, and hepatocyte expansion 74, 75. After sensing lipotoxicity-associated ceramide, the NLRP3 inflammasome induces caspase-1 cleavage, and ultimately leads to pyroptosis at the NAFL stage 76. The inflammation or fibrosis induced by pyroptosis is more serious at the stage of NASH 74, 75. A study proved that GSDMD-NT was upregulated in NAFL and showed higher levels in NASH 77. GSDMD knockout mice fed with methionine-choline deficiency showed milder steatosis and inflammation compared with WT mice. These results indicated that pyroptosis executor GSDMD-NT is responsible for the pathogenesis of NAFLD by regulating adipogenesis and secreting inflammatory cytokines 77.

Due to the serious inflammatory response and liver damage caused by the excessive intake of alcohol, alcoholic liver disease presents a very high mortality worldwide. However, owing to our poor understanding of the molecular mechanisms underlying the condition, currently, there is still no effective treatment strategy for it. It is well established that excessive uptake of alcohol is often related to different forms of cell death, including pyroptosis. Heo et al. discovered that alcohol can decrease the expression of miR-148a through forkhead box O1 (FoxO1) in hepatocytes, which leads to overexpression of thioredoxin-interacting protein and activation of NLRP3 inflammasome, eventually inducing pyroptosis in hepatocytes 78. By reducing caspase-1-induced pyroptosis, selenium-enriched *S. platensis* displays a protective role in chronic alcohol-induced liver injury 79.

Additionally, liver inflammation has been shown to be related to pyroptosis during the development of liver fibrosis. The fibrosis-related proteins are mainly derived from hepatic stellate cells, which get activated and produce collagen through pyroptosis 80. In addition to hepatic stellate cells, infiltrated eosinophils have been shown to induce secretion of pro-inflammatory cytokines IL-18 and IL-1β, or even pyroptotic cell death of hepatocytes, leading to liver fibrosis. The caspase-1 inhibitors significantly suppress this process, further suggesting that pyroptosis plays a crucial role in eosinophil-induced hepatic fibrosis 81.

The above studies indicate that inhibiting pyroptosis might be a potential therapeutic strategy for liver diseases. So, there are many researches targeting pyroptosis for the treatment of liver diseases, mainly involving two strategies: direct inhibition of NLRP3 inflammasome and restraining of downstream signaling pathways of the NLRP3 inflammasome (Table 2). Qu et al. 82 demonstrated that MCC950, which is known as an NLRP3 inhibitor, significantly alleviates bile duct ligation-induced liver fibrosis by reducing IL-18 and IL-1β expression, and suppressing neutrophil infiltration and hepatic cell death. P2X7 inhibitors prevent ATP-mediated activation of NLRP3 83. In addition to these inhibitors, some herbal extracts and ingredients can inhibit signaling pathways of pyroptosis and reduce liver damage. Zhang et al. showed that silybin significantly inhibits the assembly of NLRP3 inflammasome in mice with NAFLD 84. A study has demonstrated that dihydroquercetin can decrease the expression of P2X7 and NLRP3, and subsequently suppress cleavage of caspase-1 in an animal model of alcoholic liver steatosis 85. Liraglutide, an analog of glucagon-like peptide-1, has been shown to inhibit NLRP3 inflammasome-mediated pyroptosis and attenuate mitochondrial dysfunction, which significantly ameliorates NASH 86.

The potential effects of caspase-1 87, IL-18 88, and IL-1β 89 inhibitors have been studied to target the downstream signaling pathways of the NLRP3 inflammasome. GSDMD is the final executor, but there are very few studies targeting it. The potential effect of GSDMD inhibitors requires further investigation for the treatment of various liver diseases in the future.

### Nervous system diseases

Emerging studies imply that pyroptosis may be involved in the pathology of nervous system diseases such as ischemic stroke, Parkinson's disease (PD), and Alzheimer's disease (AD). AD is a common neurodegenerative disease that is characterized by dementia and cognitive decline. The main pathological features of AD are β-amyloid protein (Aβ) deposition in the extracellular neuritic plaque, neurofibrillary tangles due to aggregation of abnormally phosphorylated tau protein, vascular amyloidosis, and neuronal death in the brain. Aβ or hyperphosphorylated tau can activate NLRP1, AIM2, and NLRP3 inflammasome, eventually resulting in pyroptosis of neurons both *in vitro* and *in vivo* 90, 91.

PD is another neurodegenerative disorder characterized by the loss of dopaminergic neurons in the midbrain. Accumulating evidence demonstrates the involvement of pyroptosis in PD. miR-135b alleviates 1-methyl-4-phenylpyridinium-induced PD in an *in vitro* model by suppressing FoxO1-induced NLRP3 inflammasome activation and pyroptosis, which suggests that pyroptosis contributes to PD progression 92. Moreover, the long non-coding RNA HOTAIR facilitates NLRP3-mediated pyroptosis to aggravate neuronal damage in PD 93. Taken together, these studies indicate that inhibiting pyroptosis might be a novel therapeutic strategy for PD.

In addition to AD and PD, recent studies have demonstrated that pyroptosis of microglia or neurons participates in ischemic stroke. Yan et al. showed that neuronal pyroptosis is conducive to early ischemic injury through the SIRT1-ROS-tumor necrosis factor (TNF) receptor-associated factor 6 signaling pathway 94. Moreover, neuron pyroptosis may cause mitochondrial dysfunction, eventually leading to increased ROS levels and aggravated ischemic injuries. In addition, the diffusion of intracellular inflammatory factors is facilitated by GSDMD-mediated pyroptosis in astrocytes, microglia, and infiltrating macrophages, which promotes ischemic brain injury 95-97.

**Figure 4:**
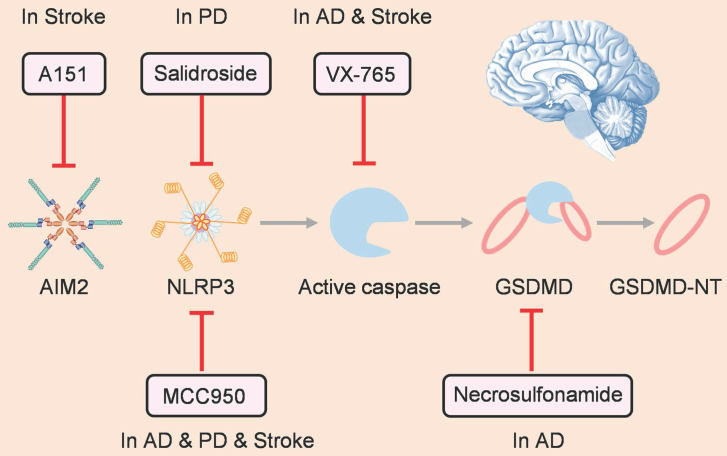
Therapeutic strategies for treating nervous system diseases by targeting pyroptosis.

As pyroptosis plays a prominent role in pathological process of nervous system diseases, many small-molecule inhibitors have been developed to target pyroptosis-related signaling pathways for treating these diseases (Figure 4). Inflammasome family members have attracted the most attention as the starting point of pyroptosis. MCC950 is a well-known selective inhibitor of NLRP3, which can alleviate the pathological progression of various nervous system diseases, such as AD 98, PD 99, and ischemic stroke 94. Furthermore, salidroside can suppress NLRP3-dependent pyroptosis to ameliorate PD 100. In addition, as an antagonist of cyclic GMP-AMP synthase and AIM2 inflammasome, A151 prevents pyroptosis of microglia and reduces infarct volume, ultimately relieving neurodeficits after ischemic stroke 96.

Downstream of the inflammasome, active caspase can cleave gasdermin protein and drive pyroptosis. Hence, caspase is another attractive target for inhibiting pyroptosis. For instance, as a caspase-1 inhibitor, VX-765 can reduce pyroptosis to alleviate injury after AD 101 and stroke 102. Gasdermin proteins are the final executors of pyroptosis, and there are drugs that target gasdermins directly. Han et al. showed that necrosulfonamide, which can inhibit GSDMD oligomerization by binding to the amino acid of C191, suppresses Aβ-triggered neuronal pyroptosis *in vivo* 91.

## Inducing cancer cell pyroptosis for cancer therapy

The mechanisms of pyroptosis in cancer cells and immune cells are different. In cancer cells, inflammasome is not necessary for pyroptosis induction and other active proteases except caspases are able to cleave gasdermins 103, in which almost all gasdermins can serve as the executors. Pyroptosis plays a vital role in tumor development and antitumor immunity, by acting as a double-edged sword that can show both tumor-promoting and tumor-suppressing effects. On the one hand, long-term chronic pyroptosis of cancer cells triggered by the adverse TME is more likely to promote cancer progression. Chronic pyroptosis triggers pro-inflammatory cytokines that facilitate the formation and maintenance of an inflammatory microenvironment for tumor growth. It has been reported that GSDME-mediated pyroptosis promotes the development of colitis-associated colorectal cancer by releasing high-mobility group box protein 1, which induces tumor cell proliferation and the expression of proliferating nuclear antigen through the ERK1/2 pathway 104. Chronic inflammation and pyroptosis also involves the development of asbestos-associated mesothelioma 105. On the other hand, acute and immense activation of pyroptosis results in numerous immune cells infiltration, which not only induces massive cancer cell death but also activates antitumor immunity to repress tumor growth 106. The antitumor immunity of pyroptosis involves many respects, which starts with the release of damage-associated molecular patterns and inflammatory cytokines that directly modulates the innate immune response, to enhance the recruitment of adaptive immune cells along with increased antigen presentation, resulting in extensive immune activation. The released inflammatory cytokines IL-1β can induce dendritic cell (DC) maturation, activate CD8^+^ T cells, and inhibit the differentiation of immunosuppressive T regulatory cells 107. IL-18 plays critical role in natural killer (NK) cell recruitment and activation, as well as Th-1 polarization 108. All of these alter the immunosuppressive microenvironment and increase tumor-infiltrating lymphocytes. Thus, inducing acute and massive cancer cell pyroptosis is a potential strategy for tumor treatment. Herein, we summarize the latest progress in pyroptosis-based cancer therapy (Table 3), and the related immune methods are also summarized (Table 4).

### GSDMD-mediated pyroptosis for cancer therapy

GSDMD was the first gasdermin discovered to be associated with pyroptosis. Shi and Kayagaki et al. showed that GSDMD participates in both canonical and non-canonical pyroptosis 8, 36. To date, it has been found that both inflammatory caspase-1/4/5/11 and apoptotic caspase-8 can cleave GSDMD to induce pyroptosis.

Simvastatin is a well-established anti-hyperlipidemic drug that inhibits 3-hydroxy-3-methylglutaryl-coenzyme A reductase to reduce cholesterol levels. Recently, Wang et al. demonstrated that simvastatin can activate NLRP3-caspase-1 pathway to induce pyroptosis in non-small cell lung cancer (NSCLC) cell lines and mouse models 109. Inhibition of pyroptosis reduced the effects of simvastatin on cancer cell viability and mobility. These data suggest that the anti-hyperlipidemic drug simvastatin may serve as a novel therapeutic agent for NSCLC *via* pyroptosis. Qiao et al. reported that 2-(anaphthoyl) ethyl-trimethylammonium iodide (a-NETA) induces pyroptosis of epithelial ovarian cancer cells *via* the caspase-4/GSDMD pathway 110. The cytotoxic effect of a-NETA was strongly blocked by knockdown of either GSDMD or caspase-4 in ovarian cancer cells. Treatment with a-NETA significantly decreased the size of the epithelial ovarian tumors *in vivo*. These results imply that a-NETA may be a promising antitumor molecule for epithelial ovarian cancer therapy through pyroptosis.

Owing to their high self-renewal and clonogenic capacity, cancer stem cells (CSCs) are regarded as the root of tumors. However, due to drug resistance, the current therapies fail to eradicate colorectal CSCs effectively. The phenotype of CSCs and their resistance to chemotherapy drugs are related to C-X-C motif chemokine receptor 4 (CXCR4) overexpression in colorectal cancer. Based on this fact, Serna et al. constructed a self-assembling toxin nanoparticle, in which the CXCR4 ligand T22 was fused with the therapeutic material diphtheria toxin (DITOX) (T22-DITOX-H6) 125. T22 endows the specificity of toxin nanoparticles to target and kill CXCR4^+^-CSCs. Protein synthesis was hindered by DITOX, which eventually led to pyroptotic cell death. T22-DITOX-H6 also showed greater inhibition of tumor growth compared to that in the control group *in vivo*. Thus, owing to the specific CXCR4^+^ targeting and effective cytotoxicity of DITOX, this nanoparticle can efficiently eliminate apoptotic-resistant CXCR4^+^ colorectal CSCs through pyroptosis, demonstrating a promising method for colorectal cancer therapy.

To a certain degree, cellular survival depends on ion homeostasis. Altering the concentration of a specific ion is usually used as a strategy to trigger different forms of cell death. Nevertheless, because the ion balance is tightly regulated by cells, the investigation of certain ions influence on cells in a controlled manner has been obstructed. Specific hybrid metal-organic framework (MOF) nanoparticles serve as a promising candidate for transporting ions stealthily into cells and releasing an overdose of ions in a controlled manner. Ploetz et al. designed a lipid-coated MIL-100 consisting of ferric ions and trimesic acid (Lip-MOFs), to transport high amounts of iron ions into cells 111. The coated lipid not only prevents cellular recognition of the ions on the MOF surface, but also facilitates cellular uptake *via* endocytosis. After uptake, the Lip-MOFs transfer to lysosomes and then degrade into trimesic acids and Fe^3+^ ions by means of pH-dependent and cysteine-involved reduction. Lysosomal rupture and subsequent pyroptosis are triggered by large amounts of Fe^3+^ ions. The reduced expression of full-length GSDMD and increased release of IL-1β observed in this study demonstrated that pyroptosis was the dominant cell death mode. This protective ion delivery and controlled release to cells may pave the way for future applications of similar nanostructures that may be used to eliminate tumor cells in the acidic tumor environment by means of pyroptosis and elicit an immune response simultaneously. In addition, iron was also reported to induce a GSDME-dependent pyroptosis 126. Iron has been shown to trigger oxidative stress by elevating ROS. On the one hand, ROS can activate the NLRP3 inflammasome and then induce GSDMD-dependent canonical pyroptosis; on the other hand, iron enhanced ROS can cause the oxidation of the mitochondrial outer membrane protein Tom20. Oxidized Tom20 recruits Bax to mitochondria, which promotes the release of cytochrome c to activate caspase-3, eventually triggering pyroptosis by inducing GSDME cleavage. Hence, iron can induce GSDMD- or GSDME-mediated pyroptosis depending on the cell context.

As an inflammatory form of PCD, pyroptosis is a promising strategy for fighting against cancer. In an attempt to reduce side effects and achieve non-invasiveness, Wu et al. designed a series of membrane-anchoring photosensitizers to induce pyroptosis for cancer cell ablation 112. 1,1,2,2-tetraphe-nylethene-benzo[c] 1,2,5 thiadiazole-2- (diphenyl methylene) malononitrile (TBD) and phenyl rings (TBD-R) were conjugated with cationic chains to obtain aggregation-induced emission photosensitizers. Upon light irradiation, the produced ROS led to direct damage to the cell membrane and ablation of cancer cells. Along with the increase of the membrane-anchoring capability of TBD-R, pyroptosis gradually became the dominant cell death mode. To uncover the mechanism of TBD-R-initiated pyroptotic cell death, the study also evaluated the protein expression of caspase-1 and GSDMD in 4T1 cells after different treatments. The results showed that caspase-1 activation and GSDMD cleavage were enhanced upon photodynamic therapy with TBD-R. Together, this study provides a pyroptosis-based and photo-activated powerful approach for cancer cell removal.

**Figure 5:**
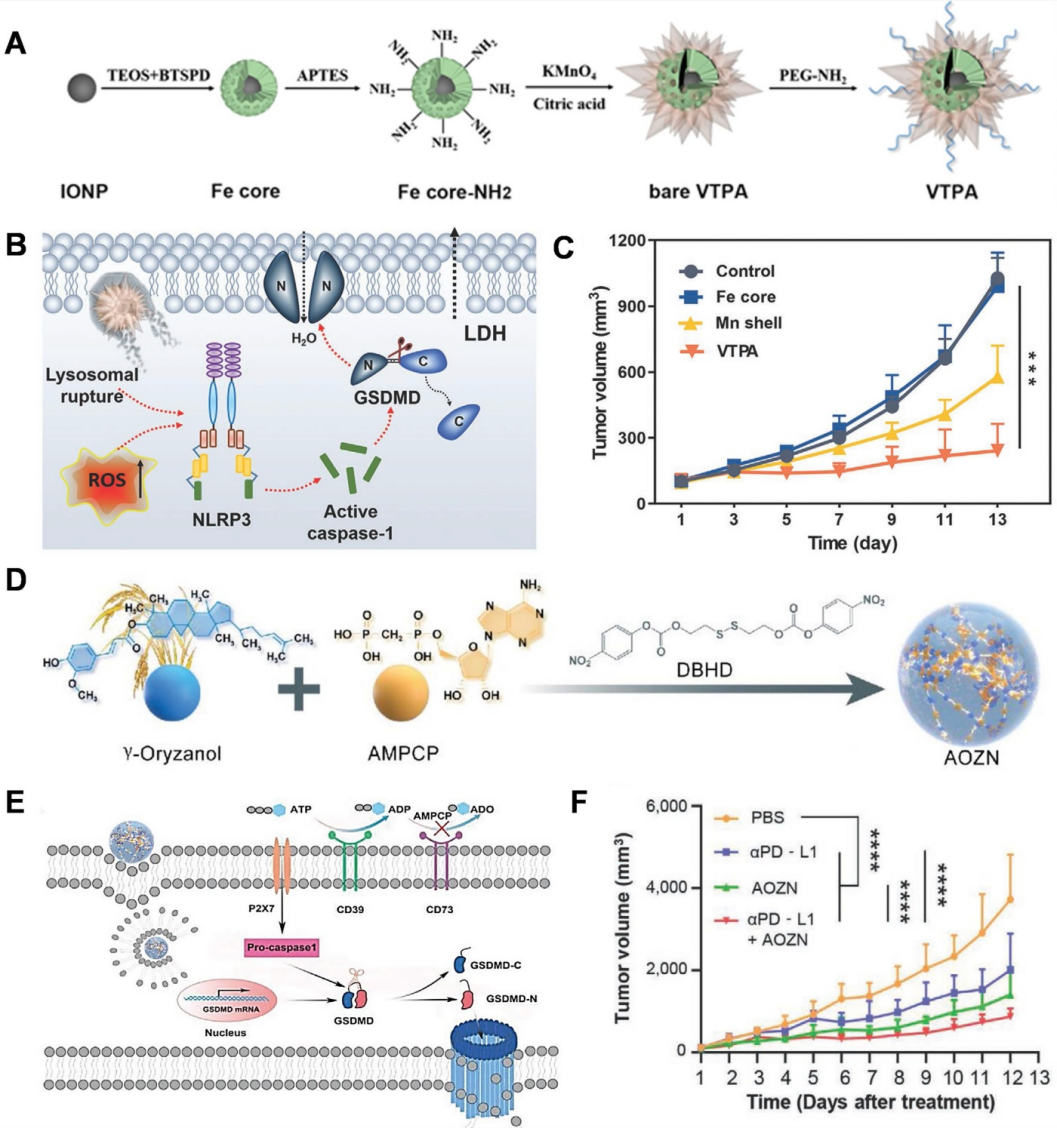
GSDMD-mediated pyroptosis for cancer therapy. (A) Schematic presentation of the designed virus-spike tumor-activatable pyroptotic agent (VTPA). (B) The molecular mechanism of VTPA triggered pyroptosis in tumor cells. (C) Changes in the tumor volume after different treatments. (D) Schematic illustration of designed nanomicelles loaded with AMPCP and Orz (AOZN) for cancer immunotherapy. (E) The mechanism of pyroptosis induced by AOZN. (F) Tumor growth curves after different treatments. Adapted with permission from 113, copyright 2021 John Wiley and Sons, and 114, copyright 2021 John Wiley and Sons.

The lack of tumor-specific pyroptotic agents *in vivo* impedes the actual applications of pyroptosis-based cancer therapy. Nadeem et al. reported the development of a virus-spike tumor-activatable pyroptotic agent (VTPA) for cancer-specific therapy 113. The VTPA consists of a manganese dioxide spiky structure and an organosilica-coated iron oxide nanoparticle (IONP) core (Figure 5A). Protrusions facilitate lysosomal rupture, following which the tumor overexpressed glutathione (GSH) triggers the degradation of VPTA to release Mn ions and IONPs for rapid and persistent ROS generation, which synergistically activates the NLRP3/caspase-1/GSDMD signaling pathway for pyroptosis (Figure 5B). Moreover, VTPA showed excellent tumor growth inhibition *via* pyroptosis *in vivo* (Figure 5C). This study provides a tumor-activatable and nanostructure-dependent pyroptotic agent, highlighting a novel direction for the development of next-generation cancer-specific pyroptotic nanomedicine in the future.

With limited T-cell responses, it is challenging to overcome innate or adaptive resistance to immune checkpoint inhibitor therapy in solid tumors. As an inflammatory form of PCD, pyroptosis is a promising strategy for enhancing cancer immunotherapy. Xiong et al. designed a GSH-responsive nanomicelles prodrug, composed of the adenosine inhibitor α, β-methylene adenosine 5' diphosphate (AMPCP) and the epigenetic modulator γ-oryzanol (Orz) for tumor therapy, which they termed as AOZN (Figure 5D) 114. When AOZN reaches the tumor site, high GSH in the TME triggers AMPCP and Orz release. The DNA methyltransferase inhibitor Orz can upregulate the expression of GSDMD, AMPCP acts as an ecto-5′-nucleotidase inhibitor to reduce adenosine levels and increase ATP accumulation, subsequently initiating NLRP3 inflammasome assembly and caspase-1 activation. Active caspase-1 directly cleaves GSDMD and induces pyroptosis in tumor cells (Figure 5E). Moreover, Orz and AMPCP synergistically combat the immunosuppressive TME (ITME). After treatment with AOZN, a more marked increase in CD8- and CD4-positive T cells was observed in the tumor tissue, while there is a significant decrease in the frequencies of regulatory T cells and CD8 T cell exhaustion in the AOZN group, as compared to those in the control group. Additionally, Orz can sensitize tumors to anti-programmed death-ligand 1 (PD-L1) therapy by increasing the expression of PD-L1 (Figure 5F). In summary, this work proposes a promising strategy to enhance cancer immunotherapy and overcome the resistance to immune checkpoint blockers.

### GSDME-based pyroptosis for cancer therapy

The expression of GSDME varies in different cancers and is mainly activated by apoptotic caspase-3 and caspase-8. Chemotherapy can activate caspase-3 to trigger pyroptosis in GSDME-expressing cancer cells 16, 39. Zhang et al. showed that the chemotherapeutic drug paclitaxel can trigger pyroptosis in A549 cells, which is closely related to the levels of activated caspase-3 and GSDME-NT 115. Compared to paclitaxel, cisplatin induced more severe pyroptosis in NSCLC cells, indicating that cisplatin may have more advantages than other drugs for the treatment of tumors with high GSDME expression. In addition to cisplatin, lobaplatin is one of the third-generation antitumor platinum that has stronger antitumor effects but fewer side effects. However, the inflammatory characteristics of lobaplatin in tumor treatment have not been reported. Yu et al. showed that lobaplatin induced ROS elevation and c-Jun N-terminal kinase phosphorylation in HT-29 and HCT116 cells, which further recruit Bax to the mitochondria, and thereby, stimulate the release of cytochrome c, followed by caspase-3/9 activation and GSDME cleavage, eventually triggering pyroptosis 116. This study showed that GSDME-mediated pyroptosis is a novel mechanism for eradicating cancer cells using lobaplatin, which is of great significance for clinical applications.

In addition to classical chemotherapy drugs, arsenic trioxide (As_2_O_3_) can accelerate the differentiation of viable cancer cells and reduce the risk of metastasis, which partly achieves better treatment responses with lower recurrence rates than traditional drugs. However, it is challenging to realize effective As_2_O_3_ accumulation inside a solid tumor with few systemic toxicities. To address this issue, Hu et al. designed a triblock copolymer monomethoxy (polyethylene glycol)-poly (d, l-lactide-co-glycolide)-poly (l-lysine) (mPEG-*b*-PLGA-*b*-PLL) nano-drug system to deliver As_2_O_3_ (As_2_O_3_-NPs). After the As_2_O_3_-NPs are internalized by tumor cells, the As_2_O_3_ is released into the cytoplasm and GSDME is cleaved following caspase-3 activation. The cleaved GSDME N-domains form membrane pores, eventually leading to pyroptosis. In *vivo* antitumor study showed that As_2_O_3_ moderately inhibited tumor growth, while As_2_O_3_-NPs substantially reduced tumor growth. As_2_O_3_-NPs treatment resulted in an increase in the protein levels of cleaved caspase-3 and GSDME-NT, with a decrease in those of Dnmt1, Dnmt3a, and Dnmt3b, thus uncovering the mechanism of the antitumor activity of As_2_O_3_-NPs 117. These data provide a new vision and strategy for future hepatocellular carcinoma therapy based on pyroptosis mediated by As_2_O_3_.

**Figure 6:**
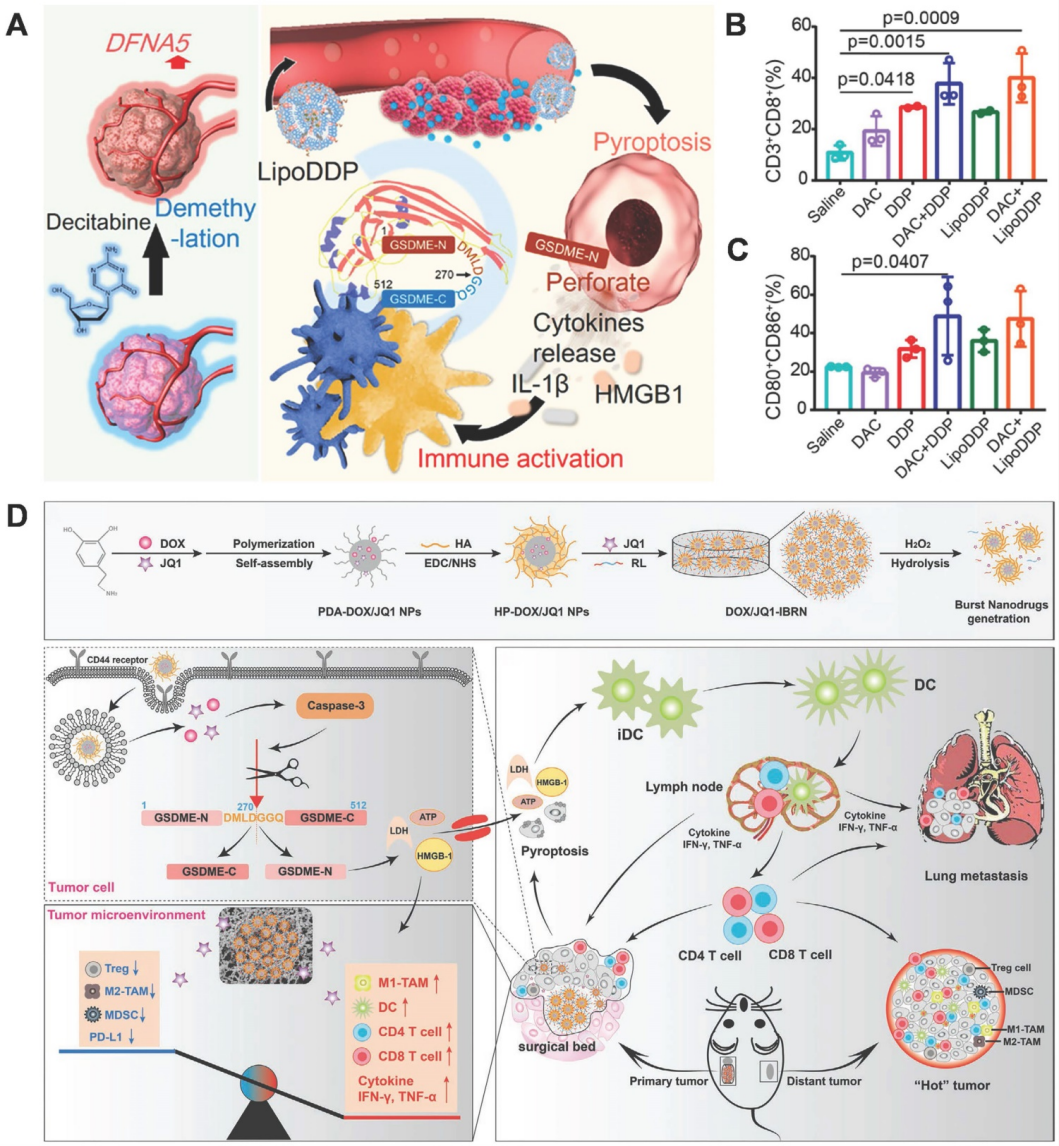
GSDME-mediated pyroptosis for cancer therapy. (A) Schematic illustration of the demethylation and immune activation process mediated by decitabine and LipoDDP *via* pyroptosis. (B) Quantification of CD4^+^ and CD8^+^ T cell-gating on CD3^+^ cells in the tumors. (C) Statistical analysis of CD80^+^CD86^+^ cell-gating on CD11c^+^ cells within tumor-draining lymph nodes. (D) Illustration of the DOX/JQ1-IBRN for post-surgical tumor treatment, involving pyroptosis of tumor cells, conversion of the ITME, and cascade activation of immunity. Adapted with permission from 118, copyright 2019 ACS, and 119, copyright 2020 John Wiley and Sons.

As mentioned above, the expression of GSDME in cancer cells varies. It is silenced in some types of tumors due to the hypermethylation of the GSDME/DFN59 gene; owing to this, GSDME-mediated pyroptosis is absent in these tumors. Fan et al. developed a strategy of combining chemotherapy with DNA demethylation to trigger cancer cell pyroptosis, which amplifies the immune effect to further eliminate tumors *via* immune therapy 118. Pretreatment of tumor cells with decitabine (DAC), a commonly used DNA methyltransferase inhibitor, resulted in the up-regulation of DFNA5 expression. Subsequently, cisplatin-loaded nanoliposome (LipoDDP) was used to activate caspase-3 and induce pyroptosis in DAC-treated tumor cells (Figure 6A). Based on its performance in terms of antitumor activities and metastasis inhibition, this combined strategy triggered the immunological effects of chemotherapy and provided a novel insight into tumor immunotherapy (Figure 6B-C).

Residual microscopic lesions after surgery and the ITME contribute to a high rate of post-operative tumor recurrence and metastasis (TRM). Drug-loaded scaffolds have the potential to inhibit TRM, but the actual therapeutic effects are limited by the ITME and untargeted toxicity from non-selective drug release. Zhao et al. constructed an implantable bio-responsive nanoarray (IBRN) to reprogram the ITME and achieve accurate tumor targeting in a controlled manner, for effective post-operative tumor therapy and TRM prevention. The chemotherapeutic DOX and epigenetic modulator JQ1 are packaged into hyaluronic acid-modified polydopamine nanoparticles, which are then linked by a ROS-responsive linker to obtain a tumor-targeted nanoarray loaded with another part of JQ1 (DOX/JQ1-IBRN) (Figure 6D). Upon reaching the tumor site, high H_2_O_2_ triggers the release of JQ1 and DOX, which realize ITME modulation and induce GSDME-dependent pyroptosis, further eliciting antitumor immunity and wiping out the residual tumor completely 119. The results showed that DOX/JQ1-IBRN inhibited post-surgical TRM and prolonged survival in tumor models with low toxicity. In summary, IBRN realizes accurate tumor pyroptosis and ITME conversion to activate antitumor immunity, for effective and safe prevention of TRM, thus providing novel insights for post-operative treatment.

Pyroptosis is considered an excellent choice to promote the immune response for cancer therapy, because of its pro-inflammatory characteristics. Zhao et al. designed a biomimetic nanoparticle (BNP) by fusing a breast cancer membrane shell onto a PLGA polymeric core loaded with indocyanine green and DAC, for photo-activated cancer cell pyroptosis and cancer immunotherapy. Due to the homing capability of the cancer cell membrane, BNP can effectively accumulate in the tumor site with low immunogenicity. The loaded indocyanine green can perforate the tumor cell membrane and induce a sudden increase in cytoplasmic Ca^2+^ through near-infrared (NIR) irradiation, which activates caspase-3 by promoting the release of cytochrome c. Meanwhile, DAC inhibits DNA methylation, which is followed by upregulation of GSDME, eventually causing pyroptosis. *In vivo*, the primary and distant tumor growth was significantly repressed within 28 days of using this strategy. After BNP treatment plus photo-activation, a high percentage of CD8^+^ T cells and CD4^+^ T cells were detected in distant tumors and spleens, and a high rate of mature DCs were detected in the primary tumor and tumor-draining lymph nodes, compared to those upon carrying out other treatments, indicating that photo-activated pyroptosis further induces inspiring antitumor immunity for cancer therapy 120. Together, BNP provides a novel strategy for photo-activated cancer cell pyroptosis and robust solid tumor immunotherapy with high compatibility.

Pyroptosis can effectively eradicate cancer cells by boosting anticancer immunity; however, due to its non-selectivity, most of the current pyroptosis inducers may cause severe side effects in cancer therapy. Wang et al. first reported that the NIR fluorophore-based hemicyanine CyNH_2_ can kill cancer cells and boost antitumor immunity by inducing pyrolysis. To realize the tumor-specificity of CyNH_2_, cancer cells high expressing NAD(P)H: quinone oxidoreductase isozyme 1 (NQO1)-responsive CyNH_2_ (NCyNH_2_) were designed to trigger pyroptosis and further activate systemic antitumor immunity. In addition, NCyNH_2_ was further encapsulated in PEG-*b*-PLGA as a theranostic nanocarrier (NCyNP) for systemic administration and fluorescence imaging *in vivo*. NCyNPs combined with anti-programmed death-1 (PD-1) enhance the antitumor effect and prevent tumor recurrence by producing powerful memory efficacy 120. Hence, the NIR fluorophore-based CyNH_2_ may represent a novel theranostic agent for initiating tumor pyroptosis selectively and triggering immunotherapy efficiently. To achieve higher tumor specificity, Xiao et al. designed a TME reactive ROS/GSH dual-responsive nano-prodrug loaded with photosensitizer purpurin 18 and paclitaxel (MCPP) to induce GSDME-mediated pyroptosis in cancer cells specifically. Upon laser irradiation, ROS produced by photosensitizer purpurin 18 realizes controlled release and triggers cancer cell pyroptosis with paclitaxel* via* chemo-photodynamic therapy. Pyroptotic cancer cells further initiate adaptive immunity and boost immune checkpoint blockade efficiency to prevent tumor growth and recurrence 122. This study not only provides a highly efficient strategy to induce pyroptosis in tumor cells specifically, but also resolves a challenge in immune checkpoint blockade *via* pyroptosis.

### GSDMC/B/A-related pyroptosis for cancer therapy

In addition to GSDME and GSDMD, the N-terminal domain of GSDMC/B/A also has the capacity to form pores in the cell membrane, to execute pyroptosis. Hou et al. showed that nuclear PD-L1 (nPD-L1) switches TNF-α-induced apoptosis to GSDMC-mediated pyroptosis in cancer cells 42. Under hypoxic stress, p-Stat3 interacts with PD-L1 and promotes its nuclear translocation, which transcriptionally activates GSDMC expression. After TNF-α treatment, active caspase-8 cleaves GSDMC to release its N-terminal domain, which forms pores on the cell membrane, eventually inducing pyroptosis. In addition, nPD-L1-switched pyroptosis is required for tumor necrosis *in vivo*. In brief, this study found a novel function of PD-L1 and identified that caspase-8/GSDMC mediates the pyroptosis pathway in cancer cells, which facilitates tumor necrosis.

**Figure 7:**
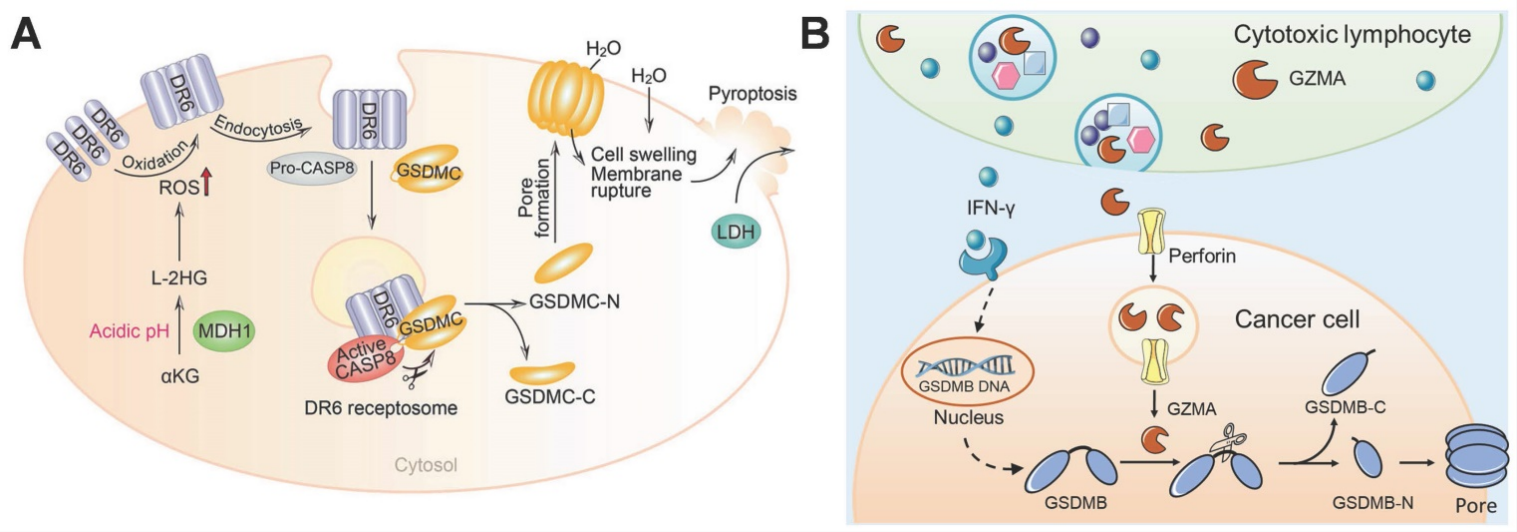
GSDMC/B-based pyroptosis for cancer therapy. (A) The working model of α-KG-induced pyroptosis *via* GSDMC. Adapted with permission from 123, copyright 2021 Springer Nature. (B) Cytotoxic lymphocyte-derived GZMA cleaves GSDMB in cancer cells to perforate the cell membrane and induce pyroptosis.

Metabolic homeostasis and metabolites affect cell fate. α-ketoglutarate (α-KG) is an essential metabolite in the tricarboxylic acid cycle that plays important roles in many physiological processes, such as oxidative stress reduction and cell death. Nevertheless, the role of α-KG in pyroptosis remains unknown. Zhang et al. demonstrated that dimethyl α-KG can pass through the cell membrane and induce the oxidation and endocytosis of death receptor 6 (DR6) by elevating ROS levels 123. After DR6 internalization, both pro-caspase-8 and GSDMC are recruited to the DR6 receptosome, where active caspase-8 cleaves the GSDMC, finally resulting in pyroptosis (Figure 7A). α-KG-induced pyroptosis has been shown to be sufficient for inhibiting tumor growth and metastasis *in vivo*. Fascinatingly, in an acidic environment, α-KG can be reduced to L-2-hydroxyglutarate (L-2HG) by the metabolic enzyme malate dehydrogenase 1, which further boosts ROS and promotes pyroptosis. Treatment with lactic acid produces more L-2HG because of the improved acidic environment, which turns pyroptosis-resistant cancer cells into pyroptosis-sensitive cancer cells. Collectively, this study links metabolites with the pyroptosis pathway and illustrates how α-KG triggers DR6 endocytosis to bring about caspase-8/GSDMC-mediated pyroptosis, and thus, has application in cancer therapy.

During the period when apoptosis was considered the dominant form of PCD, granzymes were thought to kill target cells by means of apoptosis. However, the discovery of pyroptosis has updated our understanding of PCD. It is important to explore whether gasdermin proteins respond to granzymes and induce pyroptosis. Zhou et al. demonstrated that cytotoxic T lymphocyte- or NK cell-derived GZMA can cleave GSDMB to release its pore-forming N-terminal domain for pyroptosis induction 50. Moreover, interferon-γ increased the expression of GSDMB to further promote pyroptosis (Figure 7B). Heterologous overexpression of GZMA-cleavable human GSDMB in mouse cancer cells accelerates the elimination of tumors *in vivo*. This study identified a novel killing mechanism of cytotoxic lymphocytes through gasdermin-mediated pyroptosis, which ensures sufficient antitumor immunity.

Bioorthogonal chemistry presents a wonderful strategy for investigating many biological processes, such as immunity and cell death in live animals. Wang et al. constructed a nano-bioorthogonal chemical system, in which gasdermin A3 (GA3) was linked to nanoparticles *via* the trimethylsilyl ether linker (NP-GA3), and this linker could be cleaved by a cancer-imaging probe phenylalanine trifluoroborate (Phe-BF3) 124. When HeLa and EMT6 cells were treated with NP-GA3 and Phe-BF3, the cells showed obvious pyroptotic morphology. It should be noted that only a small fraction of the tumor cells undergoing pyroptosis could erase the entire tumor, which implies the role of the immune system. Furthermore, only one round of injection of NP-GA3 and Phe-BF3 could not prevent tumor growth. In contrast, tumor growth was markedly inhibited by one round of injection plus anti-PD-1 therapy, which demonstrates that inflammation induced by pyroptosis can trigger robust antitumor immunity and synergize with immune checkpoint blockade for tumor immunotherapy.

## Conclusion

Pyroptosis is a form of inflammatory PCD mediated by gasdermin proteins, which are often activated by caspases. Initially, researchers focused on the role of inflammatory caspases (caspases-1/4/5/11) in pyroptosis. Afterwards, researchers have found that apoptotic caspases (caspases-3/6/8) are also involved in the process of pyroptosis. Moreover, recent studies have shown that GZMA/GZMB can trigger pyroptosis as well as caspases. These studies renovate our understanding of pyroptosis. Future research will continue to update the novel and precise activation modes of pyroptosis, for instance, which caspases or other factors mediate the cleavage of the GSDMA.

Generally, GSDMD cleavage by caspase-1 *via* the canonical inflammasome pathway plays an important role in host defense against pathogen infections. However, excessive inflammatory responses and cell death caused by pyroptosis may be involved in various diseases, such as cardiovascular diseases, nervous system diseases, and liver diseases. Hence, many studies have focused on inhibiting pyroptosis to treat these diseases by targeting NLRP3, caspase-1, or GSDMD. VX-765 is a safe and effective inhibitor of caspase-1 that has been proved to be well tolerated in phase II clinical trial in patients with partial epilepsy. Therefore, VX-765 is a clinical-grade drug that could potentially be used in other pyroptosis-related diseases. Another anti-pyroptotic drug currently in clinical application is lncRNA NBR2, which regulates endothelial pyroptosis by targeting GSDMD in sepsis. Nevertheless, due to non-specificity, many current inhibitors may result in unexpected side effects. Further research is needed to improve the specificity of pyroptotic inhibitors. In addition, current therapeutic targets for the treatment of these diseases mainly focus on canonical inflammasome signaling, that is, the caspase-1/GSDMD pathway. It should be further investigated whether the non-canonical inflammasome pathway or apoptotic caspases-mediated pathway of pyroptosis is implicated in these inflammatory diseases and can serve as therapeutic targets.

Although pyroptosis produces pathogenic effects on inflammatory diseases, as a pro-inflammatory cell death, it also paves a new way for cancer clearance by deliberate induction of pyroptotic cell death and intense antitumor immunity. In cancer, pyroptosis has been shown to be triggered by almost all signaling pathways in which GSDME, GSDMD, GSDMC, or GSDMB serve as the executors. Induction of pyroptosis to eliminate tumor cells has become a promising strategy for the treatment of tumor by boosting antitumor immunity. However, gasdermins are also expressed in normal tissues. Extensive pyroptosis may cause severe damage to the normal tissues. Hence, there are several points to be noted for pyroptosis-based cancer therapy. Firstly, how to specifically induce pyroptosis in cancer cells but not in normal cells for cancer therapy. Some studies have designed TME responsive nanodrug to induce pyroptosis in cancer cells specifically. Secondly, how to visualize pyroptosis *in vivo,* to further improve its accuracy. Future studies are urgently needed to develop more precise and tumor-specific pyroptotic treatments, and more clinical trials are needed to explore the potential application of pyroptosis-based cancer therapy.

## Figures and Tables

**Table 1:** Potential strategies targeting pyroptosis to treat cardiovascular diseases

Targets	Agents	Disease model	Findings	Ref.
NLRP3	INF4E	IRI in mouse	Reduces infarct size at 60 min	60
	OLT1177	IRI in mouse	Limits infarct size and preserves left ventricular contractile function	61
	6673-34-0	IRI in mouse	Limits the infarct size	62
	MCC950	MI in pig	Reduces infarct size and preserves cardiac function	63
	Colchicine	MI in mouse	Improves chronic cardiac function and survival	66
	Melatonin	Atherosclerosis in mouse	Reduces the atherosclerotic plaque in aorta	67
	PDA@M	IRI in rat	Decreases the infarct size and improves the cardiac function	68
Caspase-1	VX-765	IRI in rat	Reduces infarction and preserves ventricular function	69

**Table 2:** Targeting the signaling pathways of pyroptosis to treat liver diseases

Targets	Agents	Disease	Mechanism	Ref.
NLRP3	MCC550	Liver fibrosis	Reduces expression of IL-1β and IL-18, and suppresses neutrophil infiltration and hepatic cell death	82
	P2X7 inhibitor	Liver disease	Prevents ATP-mediated activation of NLRP3	83
	Silybin	NAFLD	Inhibits assembly of NLRP3 inflammasome	84
	Dihydroquercetin	Alcoholic liver disease	Decreases expression of P2X7and NLRP3, and suppresses cleavage of caspase-1	85
	Liraglutide	NAFLD	Inhibits the NLRP3 inflammasome and pyroptosis activation	86
Downstream of NLRP3	Caspase-1 inhibitor	Liver disease	Prevents caspase-1-dependent cell death	87
	Rosiglitazone	NAFLD	Inhibits production of hepatic IL-18	88
	IL-1β receptor antagonist	Liver fibrosis	Block IL-1-mediated inflammation in selective liver fibrotic disease	89

**Table 3:** Inducing cancer cell pyroptosis for cancer therapy

Strategy	Cancer types	Mechanism	Ref.
GSDMD-mediated pyroptosis for cancer therapy			
Simvastatin	NSCLC	NLRP3/caspase-1/GSDMD	109
a-NETA	Ovarian cancer	Caspase-4/GSDMD	110
Lip-MOF	Cervical cancer	Caspase/GSDMD	111
TBD-R	Breast cancer, cervix carcinoma, and glioblastoma	ROS/caspase-1/GSDMD	112
VTPA	Breast cancer	Lysosomal rupture and ROS/NLRP3/caspase-1/GSDMD	113
AMPCP	Melanoma	ATP/NLRP3/caspase-1/GSDMD	114
GSDME-mediated pyroptosis for cancer therapy			
Paclitaxel, cisplatin	Lung cancer	Caspase-3/GSDME	115
Lobaplatin	Colon cancer	ROS and pJNK/ Bax/ Cytochrome c/Caspase-3/9/GSDME	116
As_2_O_3_-NPs	Hepatocellular carcinoma	Caspase-3/GSDME	117
DAC+LipoDDP	Breast cancer	Caspase-3/GSDME	118
DOX/JQ1-IBRN	Breast cancer	Caspase-3/GSDME	119
BNP	Breast cancer	Ca^2+^/Cytochrome c/ Caspase-3/GSDME	120
NCyNP	Breast, lung, and cervical cancers.	CyNH_2_/Cytochrome c/ Caspase-3/GSDME	121
MCPP	Colon cancer	ROS/ Caspase-3/GSDME	122
GSDMC/B/A-mediated pyroptosis for cancer therapy			
α-KG	Cervical cancer and melanoma	ROS/DR6/Caspase-8/GSDMC	123
GSDMB	Colon cancer	GZMA/GSDMB	50
Phe-BF_3_+NP-GA3	Cervical and breast cancer	Phe-BF_3_/GSDMA3	124

**Table 4:** Summary of the strategies that induce pyroptosis for cancer therapy related to immune methods

Strategy	Cancer types	Immune response	Ref.
AMPCP	Melanoma	Remodels ITME and sensitizes tumors to anti-PD-L1 therapy	114
DAC+LipoDDP	Breast cancer	Secrets IL-1β and HMGB1, induces the DCs maturation, and increases presence of CTLs	118
DOX/JQ1-IBRN	Breast cancer	Modulates ITME, JQ1 blocks PD-L1 mediated immune evasion, and reduces Tregs	119
BNP	Breast cancer	Secrets pro-inflammatory factors to induce DC maturation and T cell activation in TDLNs	120
NCyNH2, NCyNP	Breast, lung, and cervical cancers.	Promotes CTLs infiltration in TME and DCs maturation in TDLNs, synergizes with αPD-1 to induce antitumor immunity and generates an immune memory effect	121
MCPP	Colon cancer	Initiates adaptive immunity, boosts the PD-1 blockade efficiency, generates immunological memory, and prevents tumor recurrence.	122
GSDMB	Colon cancer	Promotes CTL-mediated tumor clearance when combined with αPD-1	50
Phe-BF_3_+NP-GA3	Cervical and breast cancer	Increases CD4^+^, CD8^+^, and NK cell populations, decreases Tregs and myeloid-derived suppressor cell populations	124

## Abbreviations

ASC: apoptosis-associated speck-like protein containing a caspase recruitment domain
AD: alzheimer's disease
AIM2: absent in melanoma 2
AMPCP: α, β-methylene adenosine 5' diphosphate
AOZN: AMPCP and Orz loaded nanomicelles
ATP: adenosine triphosphoric acid
Aβ: β-amyloid protein
BNP: biomimetic nanoparticle
CARD: caspase recruitment domain-containing
CSCs: cancer stem cells
CXCR4: C-X-C motif chemokine receptor 4
DAC: decitabine
DC: dendritic cell
DITOX: diphtheria toxin
DR6: death receptor 6
FoxO1: forkhead box O1
GA3: gasdermin A3
GSDMD: gasdermin D
GSDMD-NT: gasdermin D N-terminal domain
GSH: glutathione
GZMA: granzyme A
GZMB: granzyme B
IBRN: implantable bio-responsive nanoarray
IL-18: interleukin-18
IL-1β: interleukin-1β
IONPs: iron oxide nanoparticles
IRI: ischemia-reperfusion injury
ITME: immunosuppressive tumor microenvironment
LipoDDP: cisplatin loaded nanoliposome
LPS: lipopolysaccharide
LRR: leucine-rich repeats
MCPP: dual-responsive nano-prodrug loaded with photosensitizer purpurin 18 and paclitaxel
MOF: metal-organic framework
NAFL: non-alcoholic fatty liver
NAFLD: Nonalcoholic-fatty liver disease
NASH: non-alcoholic steatohepatitis
NIR: near-infrared
NK: natural killer
NLRC4: nucleotide-binding oligomerization domain-like receptor caspase recruitment domain-containing 4
NLRP1/3: nucleotide-binding oligomerization domain-like receptor pyrin domain-containing1/3
NOD: nucleotide-binding oligomerization domain
NSCLC: non-small cell lung cancer
Orz: γ-oryzanol
P2X7: P2X purinoreceptor 7
PCD: programmed cell death
PD: parkinson's disease
PDA@M: polydopamine-based biomimetic nanoplatform
PD-L1: programmed death-ligand 1
Phe-BF3: phenylalanine tri‑uoroborate
PRRs: pattern recognition receptors
PYD: pyrin domain
ROS: reactive oxygen species
SIRT1: silent information regulator factor 2 related enzyme 1
TBD: 1,1,2,2-tetraphe-nylethene-benzo[c]^1^,^2^,^5^ thiadiazole-2-(diphenyl methylene) malononitrile
TME: tumor microenvironment
TNF: tumor necrosis factor
TRM: tumor recurrence and metastasis
VTPA: virus-spike tumor-activatable pyroptotic agent
α-KG: α-ketoglutarate
α-NETA: 2-(anaphthoyl) ethyl-trimethylammonium iodide
